# HIV-1 subtype influences susceptibility and response to monotherapy with the protease inhibitor lopinavir/ritonavir

**DOI:** 10.1093/jac/dku365

**Published:** 2014-09-16

**Authors:** K. A. Sutherland, J. Ghosn, J. Gregson, J. L. Mbisa, M. L. Chaix, I. Cohen Codar, J. F. Delfraissy, C. Delaugerre, R. K. Gupta

**Affiliations:** 1 Virus Reference Department, Public Health England, London, UK; 2 Department of Infection, University College London, London, UK; 3 Université Paris Descartes, EA 7327, Faculté de Médecine site Necker, Paris, France; 4 APHP, UF de thérapeutique en Immuno Infectiologie, CHU Hotel Dieu, Paris, France; 5 Department of Medical Statistics, London School of Hygiene and Tropical Medicine, UK; 6 AbbVie Laboratory, Rungis, France; 7 AP-HP, Department of Internal Medicine, Bicetre University Hospital, Le Kremlin-Bicetre, France; 8 Virology, U941 INSERM Paris Diderot University, St Louis Hospital-APHP, Paris, France

**Keywords:** antiretroviral therapy, resistance, protease inhibitor, gag

## Abstract

**Objective:**

PI susceptibility results from a complex interplay between protease and Gag proteins, with Gag showing wide variation across HIV-1 subtypes. We explored the impact of pre-treatment susceptibility on the outcome of lopinavir/ritonavir monotherapy.

**Methods:**

Treatment-naive individuals who experienced lopinavir/ritonavir monotherapy failure from the MONARK study were matched (by subtype, viral load and baseline CD4 count) with those who achieved virological response (‘successes’). Successes were defined by viral load <400 copies/mL after week 24 and <50 copies/mL from week 48 to week 96. Full-length Gag–protease was amplified from patient samples for *in vitro* phenotypic susceptibility testing, with susceptibility expressed as fold change (FC) relative to a subtype B reference strain.

**Results:**

Baseline lopinavir susceptibility was lower in viral failures compared with viral successes, but the differences were not statistically significant (median lopinavir susceptibility: 4.4 versus 8.5, respectively, *P* = 0.17). Among CRF02_AG/G patients, there was a significant difference in lopinavir susceptibility between the two groups (7.1 versus 10.4, *P* = 0.047), while in subtype B the difference was not significant (2.7 versus 3.4, *P* = 0.13). Subtype CRF02_AG/G viruses had a median lopinavir FC of 8.7 compared with 3.1 for subtype B (*P* = 0.001).

**Conclusions:**

We report an association between reduced PI susceptibility (using full-length Gag–protease sequences) at baseline and subsequent virological failure on lopinavir/ritonavir monotherapy in antiretroviral-naive patients harbouring subtype CRF02_AG/G viruses. We speculate that this may be important in the context of suboptimal adherence in determining viral failure.

## Introduction

Efficacious, long-term HAART treatment entails both a high financial cost and a risk of significant side effects, and exploration of alternative treatment regimens is necessary. Simplification strategies have been investigated to reduce the number of antiretroviral drugs required as part of treatment regimens without compromising treatment efficacy. Boosted PI (bPI) monotherapy has been studied in a small number of randomized trials, with poorer outcomes compared with standard triple therapy when used as initial therapy in the MONARK study^1,2^ or as second-line therapy after an NNRTI first-line failure in resource-limited settings.^3,4^ When used as a maintenance regimen in patients with a substantial period of viral suppression, bPI monotherapy may be considered as a treatment option to reduce exposure to antiretrovirals and NRTI-associated toxicities with the possibility of re-intensification when needed.^5–8^

Based on an estimated 10 million HIV-1-infected patients on antiretroviral therapy^9^ and a virological failure rate of 20%,^10^ around 2 million patients worldwide qualify for PI-based second-line treatment, most likely lopinavir boosted with ritonavir. Widespread use of this class of drug raises some concerns, given our poor understanding of virological failure. Indeed, the majority of patients experiencing failure of first-line combined ART with ritonavir-boosted PIs do so without evidence of major PI-associated drug resistance by standard methods.^11,12^ Studies have provided evidence for the role of Gag in PI susceptibility^13–15^ and have been reviewed.^16^ In addition, the inclusion of full-length patient-derived Gag alongside its co-evolved protease in *in vitro* phenotypic assays has been shown to affect PI susceptibility in both treatment-naive and treatment-experienced patients.^17–19^ More recently, Env has been implicated in drug resistance following PI exposure.^20^

MONARK investigated lopinavir/ritonavir monotherapy in comparison with lopinavir/ritonavir plus two NRTIs at treatment initiation in treatment-naive patients, with the monotherapy arm showing higher rates of virological failure.^1,2^ HIV-1 subtypes B and CRF02_AG were the dominant subtypes, and a higher failure rate was observed in the latter.^21,22^ This subtype is prevalent in West Africa and is also present in migrant HIV-1-infected populations in Europe, where subtype B otherwise dominates. Given the evidence for the importance of the inclusion of full-length Gag alongside its co-evolved protease in phenotypic assays and our previous study demonstrating reduced phenotypic susceptibility in patients experiencing virological failure in the MONARK trial, we hypothesized that Gag–protease-mediated susceptibility would differ between patients failing therapy and those with viral suppression, as well as between divergent strains (subtypes).^23^ This study sought to investigate the viral determinants of treatment failure in the MONARK lopinavir/ritonavir monotherapy arm trial by studying co-evolved, full-length Gag–protease from patients achieving virological response and experiencing virological failure in phenotypic PI susceptibility assays.

## Methods

### Study participants

Based on sample availability from MONARK, we studied eight individuals with lopinavir/ritonavir monotherapy failure (Figure S1, available as Supplementary data at *JAC* Online) and to these we matched eight patients (by subtype, viral load and baseline CD4 count) who achieved virological response (‘successes’). Successes were defined by viral load <400 copies/mL after week 24 and <50 copies/mL from week 48 to week 96 (Table 1).

**Table 1.** Characteristics of study participants and virus isolates from participants

Baseline characteristics	Failures (*n* = 8)	Successes (*n* = 8)	*P* value for difference between groups
Sex, *n* (% male)	8 (100)	7 (87.5)	1
Subtype B, *n* (%)	3 (37.5)	3 (37.5)	1
VL (copies/mL), median (IQR)	53 200 (41 200–79 850)	38 650 (27 300–59 000)	0.23
CD4 (cells/mm^3^), median (IQR)	256.5 (211–297)	243 (223–336)	0.10
Viral infectivity, median (IQR)	78 (60–101)	96 (79–99)	0.29

VL, viral load.

### Amplification of full-length Gag–protease

Full-length Gag–protease was amplified from patient samples as previously described.^24^ Clonal sequencing of up to 10 viral variants for each sample was performed. The variant that most closely represented the consensus was taken forward for phenotypic testing. Protease sequences were analysed for PI resistance mutations using the Stanford Resistance Database (http://hivdb.stanford.edu/) (Table S1).^25^

### PI susceptibility and infectivity assays

PI susceptibility and infectivity were determined using previously described single-cycle assays.^17,19,24^ Briefly, 293 T cells were co-transfected with p8.9NSX+-derived test vector containing patient *gag–protease*, pMDG expressing vesicular stomatitis virus envelope glycoprotein (VSV-g) and pCSFLW expressing the firefly luciferase reporter gene with HIV-1 packaging signal. To determine PI susceptibility, transfected cells were seeded with serial dilutions of lopinavir and harvested pseudovirions used to infect fresh 293 T cells. To determine strain infectivity, transfected cells were seeded in the absence of drug. Infectivity was monitored by measuring luciferase activity 48 h after infection. All experiments were performed in duplicate.

### Statistical analysis

We compared baseline characteristics of viral failures and successes using the Mann–Whitney rank sum test for continuous variables and Fisher's exact test for categorical variables. For analyses of lopinavir susceptibility, we defined each individual's susceptibility as the geometric mean of the two measurements. We compared lopinavir susceptibility between viral failures and successes by the Mann–Whitney rank sum test, which is robust for data that are not normally distributed. To check the sensitivity of our results to the choice of analytical method, we repeated the analyses using conditional logistic regression on the matched participants using viral failure or success as the outcome variable and log lopinavir fold change (FC) as the exposure. These sensitivity analyses gave concordant results.

## Results

Analyses involved 16 participants, of whom 8 were cases (failures) and 8 were controls (successes). Ten participants harboured HIV subtype CRF02_AG or G and six harboured subtype B, with subtypes split evenly between cases and controls. No patient harboured virus with major or minor resistance mutations to lopinavir, although a number of polymorphisms were present in protease, some of which are considered consensus amino acid positions in subtype CRF02_AG and G viruses (Table S1). Baseline CD4 and viral load were marginally higher in cases, but these differences were not statistically significant (Table 1).

**Figure 1.**
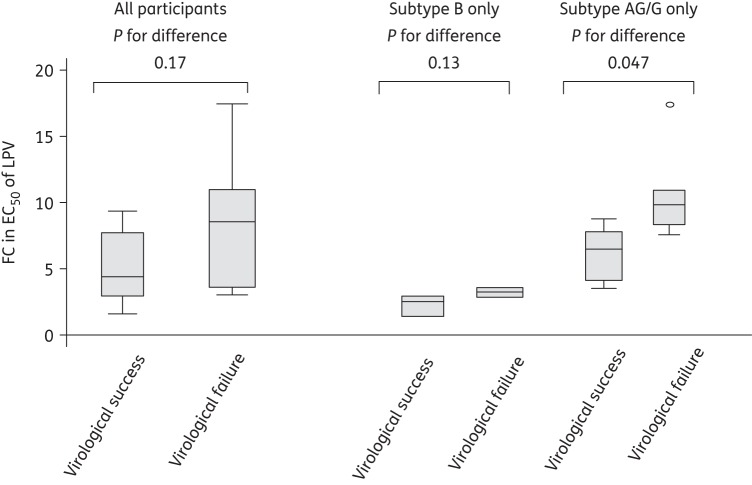
Box plots of phenotypic assay FC in EC_50_ of lopinavir (LPV) by treatment outcome.

Baseline lopinavir susceptibility was lower in viral failures than viral successes, but the differences were not statistically significant [median (IQR) lopinavir susceptibility in virological successes versus failures: 4.4 (2.9–7.7) versus 8.5 (3.6–11), *P* = 0.17, Figure 1]. When considering only the 10 CRF02_AG/G patients, there was a significant difference in lopinavir susceptibility between the two groups [median (IQR) lopinavir susceptibility in virological successes versus failures: 7.1 (4.7–8.4) versus 10.4 (8.9–11.5), *P* = 0.047, Figure 1]. When considering only subtype B patients (*n* = 6), lopinavir susceptibility appeared lower in virological failures than successes, while the difference was not significant (median lopinavir susceptibility in virological successes versus failures: 2.7 versus 3.4, *P* = 0.13) (Figure 1).

**Figure 2.**
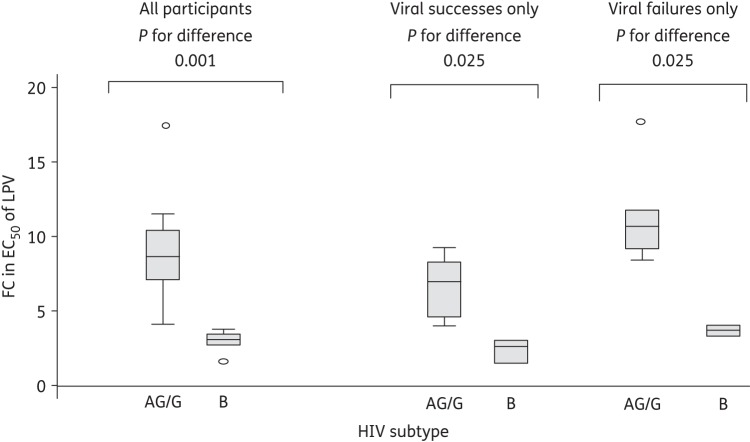
Box plots of phenotypic assay FC in EC_50_ of lopinavir (LPV) by subtype.

These data suggest there may be an inherent difference in susceptibility between subtypes AG/G and B. Subtype CRF02_AG /G viruses had a median (IQR) lopinavir FC of 8.7 (7.1–10.4), compared with 3.1 (2.7–3.4) for subtype B (*P* = 0.001) (Figure 2). This difference in lopinavir susceptibility between viruses was consistent when observed within virological successes (*P* = 0.025) or failures (*P* = 0.025).

**Figure 3.**
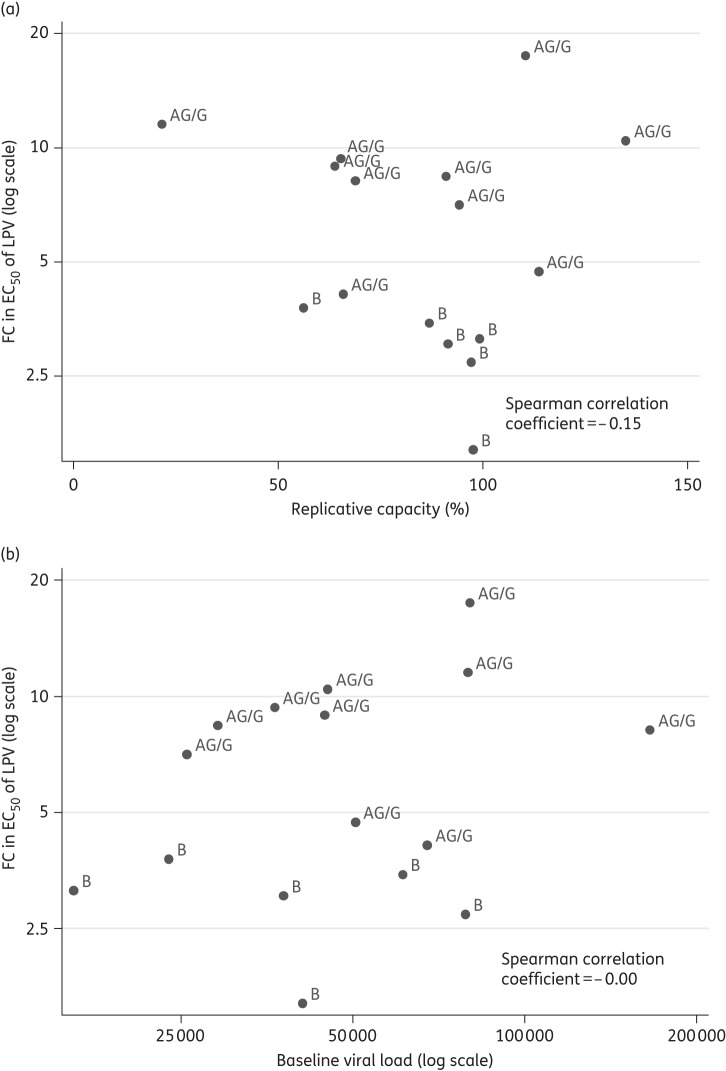
Scatter plot of FC in EC_50_ of lopinavir (LPV) versus (a) replicative capacity and (b) baseline viral load.

Finally, we also examined the relationship between drug susceptibility and replication of the viruses from each patient. Lopinavir FC showed a weak negative association with *in vitro* replication capacity (*ρ* = −0.15) (Figure 3a), and was not associated with plasma viral load (Figure 3b). Replication capacity (over a single round of infection *in vitro*) was not significantly associated with virological failure, with marginally lower levels in virological failures compared with successes [median (IQR) in virological successes versus failures: 96 (79–99) versus 78 (60–101), *P* = 0.29].

## Discussion

A previous analysis of the MONARK study suggested an association between the HIV-1 subtype AG (circulating in West Africa) and poorer outcome of lopinavir/ritonavir monotherapy, although there were confounding factors such as treatment adherence.^22^ The evaluation of a dual regimen of tenofovir plus lopinavir/ritonavir as first line in West Africa in the DAYANA trial also showed poor virological responses relative to standard NNRTI-based regimens,^26^ while the non-inferiority of another dual regimen, lopinavir/ritonavir plus lamivudine compared with a lopinavir/ritonavir-based triple regimen was demonstrated in other settings in the GARDEL trial.^27^ Here, we performed a case–control study within MONARK to test the hypothesis that baseline susceptibility, as determined by a full-length cognate Gag–protease assay, is correlated with outcome. In the primary analysis, in which subtype B and CRF02_AG/G were analysed together, we found a non-significant association between lopinavir susceptibility and failure (*P* = 0.14). In a subtype CRF02_AG/G-specific analysis, we found a significant difference between the two groups despite a relatively small sample size. Furthermore, subtype CRF02_AG/G viruses are substantially less susceptible to lopinavir than subtype B and this may have contributed to the findings of the MONARK investigators that subtype CRF02_AG patients were more likely to experience virological failure than those harbouring subtype B.

To date, phenotypic analysis using the commercial Phenosense assay (using a patient-derived protease with a subtype-mismatched Gag) had been performed on viruses derived from patients experiencing virological failure in the lopinavir/ritonavir monotherapy arm with the appearance of major PI resistance mutations.^28^ This analysis showed that in three patients with subtype CRF02_AG virus the pre-therapy EC_50_ FC was 0.57, 0.59 and 0.87 relative to the subtype B reference strain. In two subtype B-infected individuals, FCs of 1.46 and 1.49 were reported pre-therapy. The Phenosense assay result would suggest that subtype CRF02_AG viruses in MONARK are more susceptible than subtype B, in direct contradiction to our data using matched Gag and protease sequences (Figure 2 and Gupta *et al*.^17^).

However, the reduced PI susceptibility present in the variants from viral failure patients at baseline does not fully explain the subsequent treatment failure experienced. Six of the eight failure patients did initially achieve virological suppression <400 copies/mL on lopinavir/ritonavir monotherapy at week 24, before experiencing virological failure after >40 weeks of the trial. We hypothesize that PI monotherapy is potent enough to suppress viral replication with high adherence initially, but that reduced PI susceptibility lowers the tolerance or ‘buffer zone’ for subsequent suboptimal adherence. It is possible that patients with viruses demonstrating reduced PI susceptibility at baseline may be better suited to standard combined ART, which is likely to be more forgiving of reduced adherence, as three active agents are present. Alternatively, identification of these patients before treatment initiation would enable interventions to increase adherence and reduce this risk of failure. A third possibility is to closely monitor patients on PI monotherapy and intensify if low-level viraemia or viral rebound occurs.^8,29^

Limitations of our study include the relatively small sample size, the inclusion of more subtype CRF02_AG viruses in comparison with B and the possibility of viral recombination through our PCR and cloning strategy. Finally, our assay system did not incorporate the native gp160 envelope.^20^

In conclusion, we report an association between reduced PI susceptibility at baseline in the absence of known resistance mutations and subsequent virological failure on lopinavir/ritonavir monotherapy in patients harbouring subtype CRF02_AG/G viruses. This is an important finding as it indicates that it may be possible to predict treatment outcome on lopinavir/ritonavir monotherapy from baseline PI susceptibility. We hypothesize that reduced baseline PI susceptibility renders patients more vulnerable to virological rebound when their adherence is sub-optimal. This study suggests that lopinavir/ritonavir monotherapy should not be used in antiretroviral-naive patients infected with CRF02_AG. In maintenance therapy, studies are still needed to evaluate the impact of HIV-1 subtypes on virological response to PI/ritonavir monotherapy, as this regimen may be considered as a treatment option for individual patients in whom real-time viral load monitoring is available.

## Funding

This study was funded by Wellcome Trust and a grant from AbbVie Laboratories. R. K. G. is funded by a Wellcome Trust Fellowship (WT093722MA). K. A. S. was funded by Public Health England (formerly the HPA). I. C. C. was involved in interpretation of data and review of the manuscript, but final content was decided by the principal investigator.

## Transparency declarations

I. C. C. is an employee of AbbVie Laboratories and holds stock or options in AbbVie. The remaining authors have none to declare.

## Supplementary data

Table S1 and Figure S1 are available as Supplementary data at *JAC* Online (http://jac.oxfordjournals.org/).

Supplementary Data
