# Supplementary material for: HIV-1 subtype influences susceptibility and response to monotherapy with the protease inhibitor lopinavir/ritonavir
Source: J Antimicrob Chemother. 2014 Sep 16;70(1):243–8. doi: 10.1093/jac/dku365 (PMC4267506; doi:10.1093/jac/dku365)
Supplement: Supplementary Data [file supp_70_1_243__index.html]

HIV-1 subtype influences susceptibility and response to monotherapy with the protease inhibitor lopinavir/ritonavir — Supplementary Data 

# HIV-1 subtype influences susceptibility and response to monotherapy with the protease inhibitor lopinavir/ritonavir

## Supplementary Data

Supplementary Data

**Files in this Data Supplement:**

- Supplementary Data - Docx file
