# Supplementary material for: HIV-1 subtype influences susceptibility and response to monotherapy with the protease inhibitor lopinavir/ritonavir
Source: J Antimicrob Chemother. 2014 Sep 16;70(1):243–8. doi: 10.1093/jac/dku365 (PMC4267506; doi:10.1093/jac/dku365)
Supplement: Supplementary Data [file supp_dku365_dku365supp.docx]

**Supplementary data**

**Table S1. Protease polymorphisms present in viruses from all patients.** Protease polymorphisms, as determined by the Stanford resistance database, are displayed for viruses from each patient.

| **Patient** | **Treatment Outcome** | **Subtype** | **Protease polymorphisms** |
| --- | --- | --- | --- |
| 2112 | Success | B | K14RM K20I, M36I, P39L, R41K, L63P, H69K, L89M |
| 909 | Success | B | L10I, E35D, M36I, R57K, D60E, I62V, L63S, I64V, K70T, V771, I72T, I93L |
| 509 | Success | B | P39S, L63P, I64V, V77I |
| 508 | Failure | B | T31I, N37S, P39Q, I62V, L63P, V77I, I93L |
| 3204 | Failure | B | E35D, L63P, I72V, V77I, I93L |
| 2111 | Failure | B | G16E, L33V, N37Y, R57K, D60E, I62V, L63S, I64L, H69K |
| 110 | Success | AG | I13V, K14R, K20I, M36I, P39L, R41K, L63P, H69K, L89M |
| 4003 | Success | AG | I13V, K20I, M36I, R41K, H69K, K70R, L89M |
| 515 | Success | AG | T12K, I13V, K14Q, I15V, L19I, K20I, M36I, N37D, R41K, L63V, I64M, C67S, H69K, L89M |
| 1702 | Success | AG | I13V, K14R, I15V, K20I, M36I, L63P, G68E, H69K, L70R, L89M |
| 4202 | Success | G | I13V, K14R, K20I, E35Q M36I, N37D, R41K, C67E, K20R, V82I, L89M, Q92H |
| 1403 | Failure | AG | I13V, K14R, G16E, K20I, E35D, M36I, H69K, K70R, L89M |
| 1404 | Failure | AG | I13A, K20I, M36I, R41K, I64V, H69K, L89I, I93M |
| 3103 | Failure | AG | I13A, K14R, K20I, M36I, R41K, H69K, L89M |
| 3002 | Failure | AG | I13V, K14R, G16E, K20I, M36I, R41K, R57K, H69K, L89M |
| 4201 | Failure | G | I13V, K14R, K20I, E35Q, M36I, R41K, R57K, Q61N, I64M, C67E, H69K, I72V, V82I, L89M |


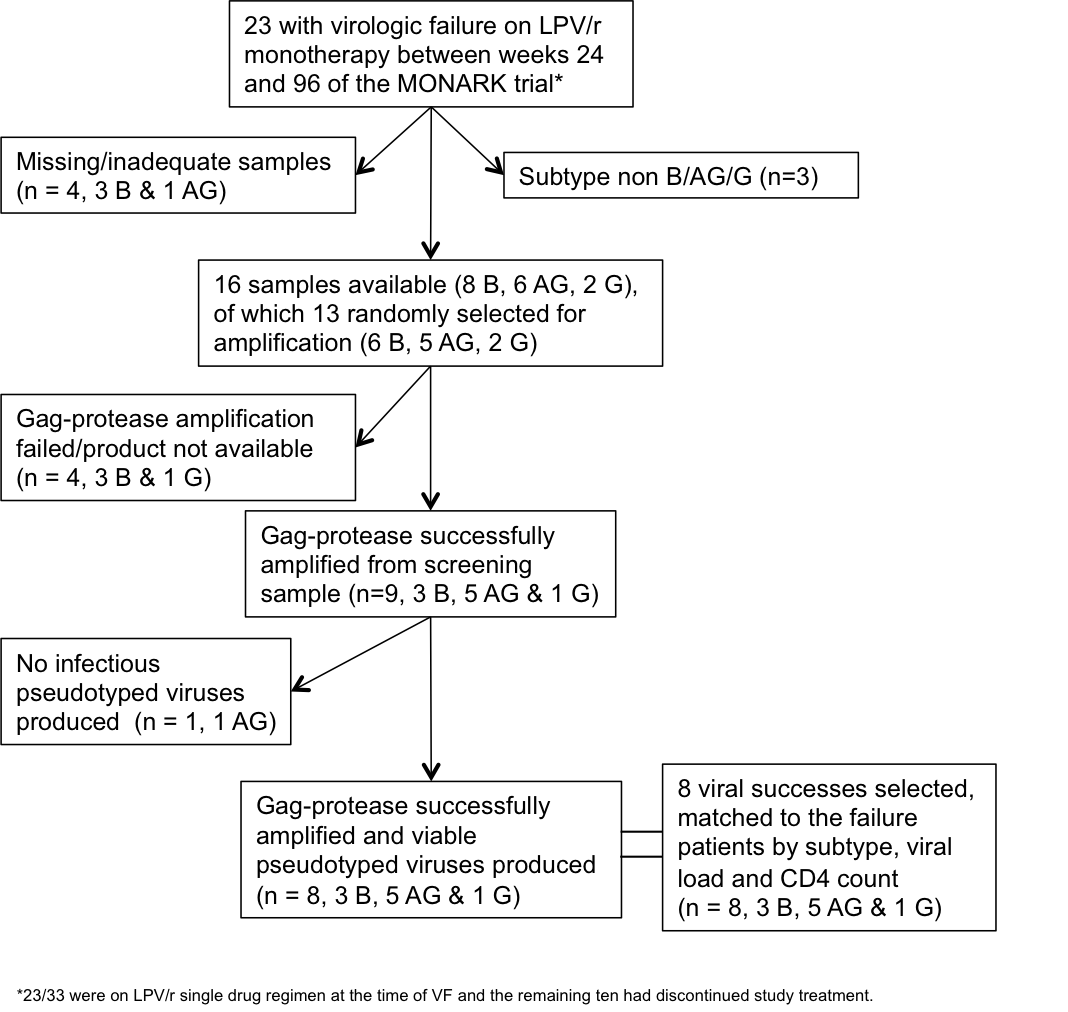


**Figure S1: Patient sample selection flow diagram.**

The selection of failure patients for this study from all patients experiencing virologic failure in the MONARK trial is shown. The number of patients with each subtype is shown at each stage. The selection of success patients, by matching to the included failure patients is shown.
